# Enhanced Binding Efficiency of Microcantilever Biosensor for the Detection of Yersinia

**DOI:** 10.3390/s19153326

**Published:** 2019-07-29

**Authors:** Xiaochen Liu, Lihao Wang, Junyuan Zhao, Yinfang Zhu, Jinling Yang, Fuhua Yang

**Affiliations:** 1 Institute of Semiconductors, Chinese Academy of Sciences, Beijing 100083, China; 2 Center of Materials Science and Optoelectronics Engineering, University of Chinese Academy of Science, Beijing 100049, China; 3 State Key Laboratory of Transducer Technology, Shanghai 200050, China

**Keywords:** microcantilever, binding efficiency, Yersinia, microfluidic system

## Abstract

A novel microcantilever sensor was batch fabricated for Yersinia detection. The microcantilever surface modification method was optimized by introducing a secondary antibody to increase the number of binding sites. A novel microfluidic platform was designed and fabricated successfully. A 30 μL solution could fully react with the microcantilever surface. Those routines enhanced the binding efficiency between the target and receptor on the microcantilever. With this novel designed microfluidic platform, the specific adsorption of 10^7^ Yersinia on the beam surface with modified F1 antibody was significantly enhanced.

## 1. Introduction

The plague, also known as the “black death,” is one of the oldest infectious diseases [1]. It caused tens of millions of deaths in ancient times, but today it still exists in more than 20 countries around the world [2,3]. Early diagnosis, especially timely detection, is essential for avoiding the plague. Yersinia is the causative agent of the plague. The Yersinia pestis YP19 antibody specifically binds to the component 1 (F1) capsular protein, which is unique to plague bacteria and exists in living and dead cells [4]. Therefore, detection of Yersinia by immunoassay is very reliable. 

A microcantilever sensor with specific receptors immobilized on its surface selectively captures the target bacteria or antigens, then converts the binding signals into mechanical signals. A cantilever beam operating in dynamic mode detects changes in the resonant frequency caused by mass loading and has ultra-high mass detection sensitivity. Many studies on cantilever-based biochemical detection have been reported, and high sensitivity and low detection limits for protein detection such as early liver cancer markers alpha fetoprotein (AFP) and hepatocyte growth factor (HGF) [5,6,7,8,9] have been achieved. However, some restrictions remain when this method is applied to detect bacteria.

For Yersinia testing using a resonant cantilever, there are mainly two constraints. First, the sensitivity of the sensor is positively correlated with the length and width of the beam, while its small dimensions decrease the capture cross-section, resulting in reduced adsorption of target molecules [10]. Therefore, increasing the adsorption efficiency of the target molecule is very important. Second, as the size of Yersinia bacteria is about 0.2 μm, steric hindrance and capillary force greatly influence the binding efficiency, so it is urgent to improve the binding efficiency.

In this paper, a novel high-sensitivity microcantilever array sensor is proposed for Yersinia detection. In order to improve the adsorption efficiency, a secondary antibody was introduced to increase the effective binding sites, and a novel microfluidic platform was successfully designed and fabricated so that a small amount of the Yersinia analyte could effectively react with the receptor F1 immobilized on the surface of the cantilever beam. 

## 2. Theory and Methods 

### 2.1. Design of Microcantilever Sensor

A rectangular beam has the advantages of simple structure and good stability; the resonance frequency *f* can be expressed as [11,12]:

(1) $$f=\frac{1}{2\pi }\sqrt{\frac{k}{m^{*}}}$$

where *k* is the stiffness coefficient and m* is the effective mass of the cantilever. The frequency change of the microcantilever, Δ*f*, is related to both the stiffness *k* and the mass change Δ*m*. For many applications the mass-change effect is dominant compared to the stiffness effect, and the stiffness term is often neglected [13,14]. The mass sensitivity Δ*m*/Δ*f* resulting from adsorption can be expressed as [15]:

(2) $$\frac{\Delta m}{\Delta f}=-\frac{2m^{*}}{f}=-0.96\pi \sqrt{\frac{0.96\rho^{3}}{E}}l^{3}w$$

where *∆m* and *∆f* are the change of the effective mass of the cantilever and its resonance frequency due to adsorption, respectively; *l* and *w* are the length and width of the microcantilever; and *ρ* and *E* are the density and Young’s modulus of silicon, respectively.

**Figure 1:**
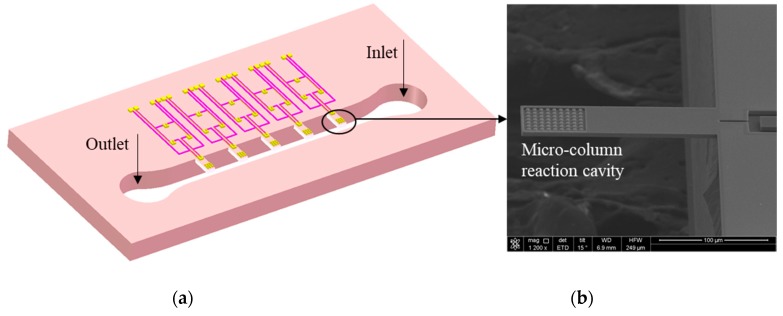
(**a**) Schematic of microcantilever biosensor and (**b**) scanning electron microscope (SEM) picture of the cantilever.

**Figure 2:**
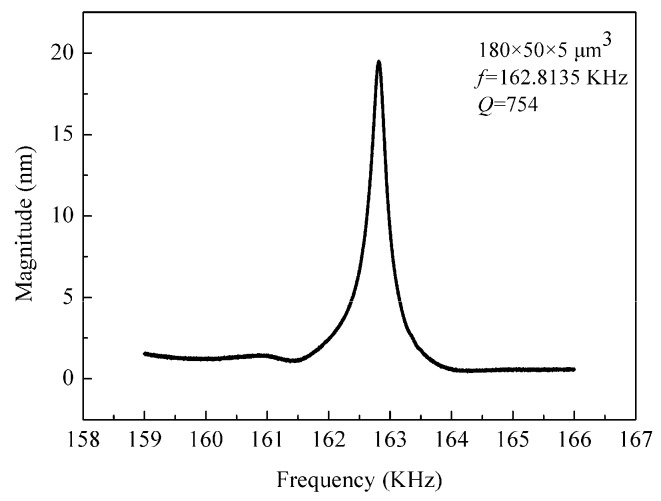
Resonance spectrum of cantilever measured in air.

The device was batch fabricated with complementary metal oxide semiconductor (CMOS) compatible processes. 4-inch (100)-oriented SOI wafers with a 5 µm-thick device layer were used. Thermo-oxygen, plasma-enhanced chemical vapor deposition (PECVD), photolithography, ion implantation, etching, and other MEMS fabrication processes were adopted [13]. The detailed fabrication processes were introduced in previous work [7]. As shown in Figure 1a, the microcantilever array sensor consists of 5 cantilever beams; at the free end of the beam, one-third of the area is the reaction cavity, and the size of the microcolumn is 3 μm × 3 μm. The inlet and outlet ports were designed on both sides of the sensor chip for better package to form microfluidic platform. Figure 1b shows scanning electron microscope (SEM) pictures of the cantilever. The cantilever was piezoelectrically driven and the response signal was detected by a laser Doppler vibration system. For a cantilever of *l* = 180 µm, *w* = 50 µm, and *h* = 5 µm, the sensitivity was 0.24 pg/Hz and the quality factor (*Q*) was 754 in air, as shown in Figure 2, which is high enough for the detection of Yersinia [16].

### 2.2. Microcantilever Surface Modification

A biochemical reaction cavity was originally designed at the free end of the cantilever to increase the adsorption area for target molecules. However, this routine did not work well for large molecules like bacteria. Instead, in this work, a fully immobilized cantilever was employed to detect bacteria.

The functionalization process of the cantilever with F1 antibody is given as follows. The silicon cantilever array was oxidized using oxygen plasma and subsequently silanized at 24 °C using 10% 3-aminopropyltriethoxysilane (APTES) solution in ethanol for 1 h. Freshly silanized cantilever was incubated at 24 °C in glutaraldehyde (GA) solution (5% v-v in deionized water) for 1 hour to form a stable bond between –NH_2_ and –CHO, thus the cantilever was able to bind with protein [17]. The cantilever array was partially immersed in the F1 antibody solution and incubated at 37 °C for 1 hour [18]. Then the lever was washed by phosphate-buffered saline (PBS; pH 7.4). The other active sites on the cantilever were then blocked by bovine serum albumin (BSA) solution at 4 °C. 

Secondary antibodies were introduced to order the F1 antibodies [19]. This was done by modifying the secondary antibodies to the surface of the cantilever before incubating the F1 antibodies, then the secondary antibodies reacted with the F1 antibodies. The microcantilever was covered by immobilized F1 antibodies and could react with Yersinia. This functionalization modification method was very stable [7]. The frequency measurement was done before and after the reaction of bacteria in solution, after cleaning and fast drying. The mass of the adsorbed bacteria could be calculated from Δ*f*. 

### 2.3. Microfluidic Platform

A microfluidic platform was fabricated to deliver the reaction solution to the biosensor and control the flow rate. It consisted of 2 polymethyl methacrylate (PMMA) plates and 2 polydimethylsiloxane (PDMS) gaskets. The sensor was located between 2 layers of PDMS, where the intermediate layers of PDMS formed microchannels by inverting molds. The upper and lower layers of PMMA were used for fixing the structure. The top plate had inlet and outlet ports.

**Figure 3:**
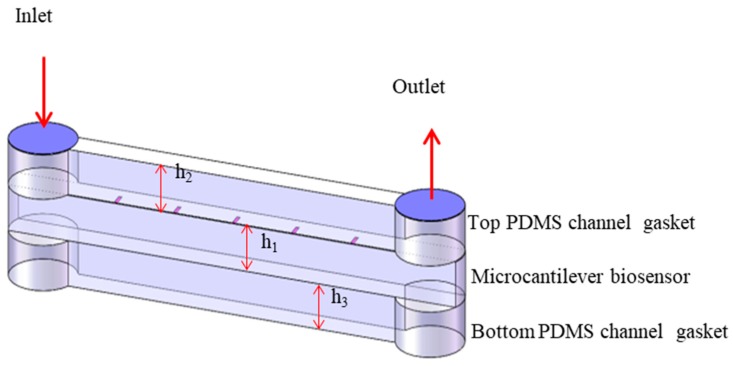
Schematic of microfluidic platform reaction chamber.

The reaction chamber of the microcantilever biochemical sensor consisted of 3 parts: the top PDMS channel gasket (*h*_1_), the microcantilever biosensor (*h*_2_), and the bottom PDMS channel gasket (*h*_3_), as shown in Figure 3. To meet the requirements of biochemical reaction, cleaning, and drying, it was necessary to ensure that the flow velocity of the fluid on the upper and lower surfaces of the cantilever was consistent. Therefore, the structural design of the reaction chamber was very important.

The Reynolds number of the fluid in the microchannel was less than 100, so the microfluidic flow in the channel was in a laminar flow regime. COMSOL finite element analysis was used to simulate the fluid movement in the reaction chamber, and the flow velocity distribution in the microchannel was obtained when *h*_2_ *= h*_3_ = [0.5:2] mm, where the flow rate *f_r_* = 1 mL/min remained unchanged.

**Figure 4:**
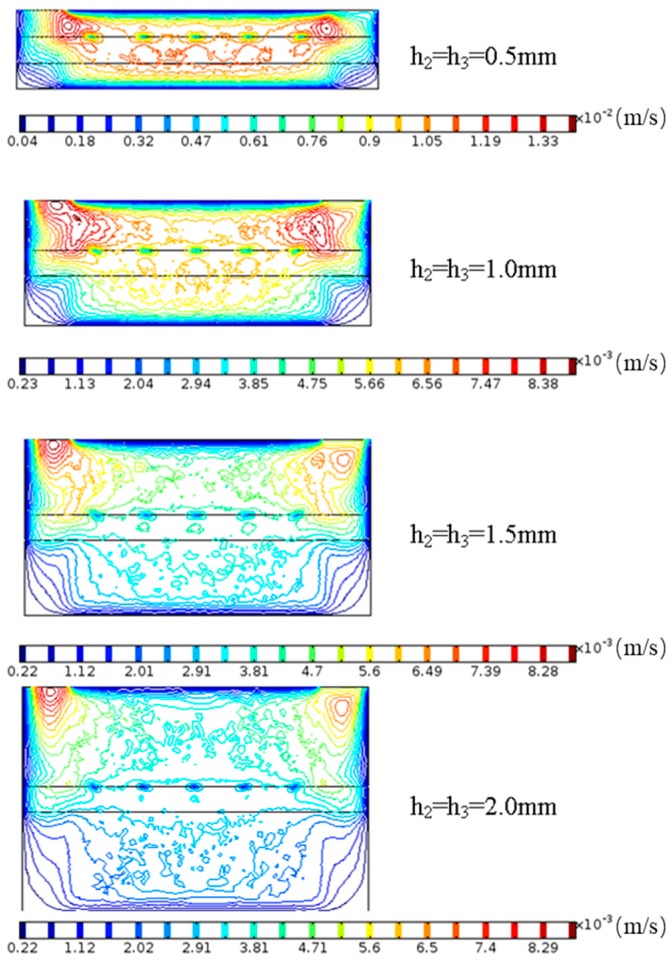
COMSOL simulation results of microchannel height (flow rate *f_r_* = 1 mL/min).

As shown in Figure 4, when *h*_2_ *= h*_3_ = 0.5 mm, the velocity of the bottom surface of the cantilever beam was more than that of the top surface, and the fluid displacement on the top surface of the cantilever beam was slow, which was not favorable for the biochemical reaction on the top surface. When *h*_2_ *= h*_3_ > 1.0 mm, the fluid flow velocity near the top and bottom surfaces of the cantilever resonator was small and the fluid replacement was slow, which was not good for cleaning and drying of the cantilever surface. In addition, the flow channel size was too large, and the probability of contact between the antigen molecule and the cantilever beam was small, which was not conducive to the biochemical reaction of the cantilever surface. When *h*_2_ *= h*_3_ = 1.0 mm, the velocity distribution of the fluid on the top and bottom surface of the cantilever resonator was basically the same, which was the optimum height of the microchannel. 

## 3. Results and Discussion

**Figure 5:**
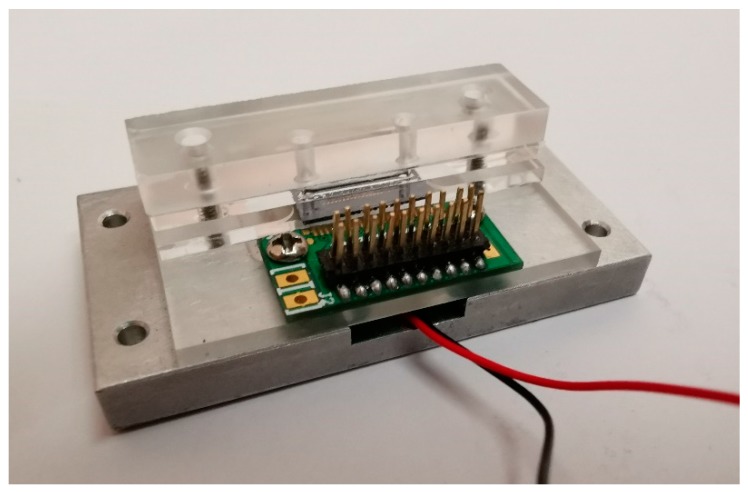
The microfluidic system.

All experiments were performed in the designed microfluidic system, as shown in Figure 5.

### 3.1. Verifying the Effect of the Secondary Antibody by Fluorescently Labeled F1 Antigen

**Figure 6:**
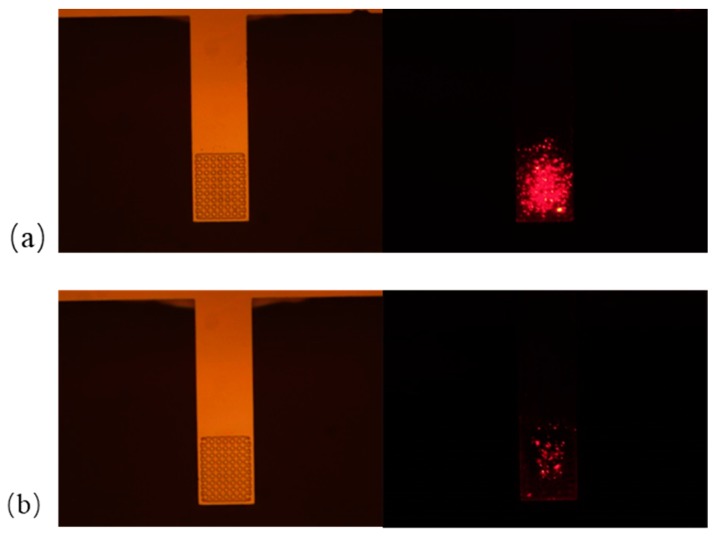
Fluorescence microscope observation results (**a**) with and (**b**) without modifying the secondary antibody to the surface of the cantilever prior to incubating the F1 antibody.

The fluorescently labeled F1 antigen was detected to verify whether the secondary antibody could increase the capture efficiency. Figure 6 depicts fluorescence microscope pictures with and without modifying the secondary antibody (1 μg/ml) to the surface of the cantilever prior to locally immobilizing the F1 antibody (1 μg/ml). The sensor with the secondary antibody modification (Figure 6a) shows a stronger fluorescence image, indicating a significant increase in antigen adsorption efficiency. This result verifies that the secondary antibody could enhance the binding sites and reaction efficiency.

In terms of the Langmuir–Hinshelwood mechanism [20], the surface adsorption reaction is related to three parameters: the concentration of “target” or “analyte” in the buffer fluid *c* (mol/L), the surface concentration [Γ]_0_ (mol/m^2^) of capture sites or ligands immobilized on a functionalized surface, and the surface concentration of the adsorbed target [Γ] (mol/m^2^). The reaction is reversible because the targets are constantly captured by ligands and can constantly dissociate at a low rate.

**Figure 7:**
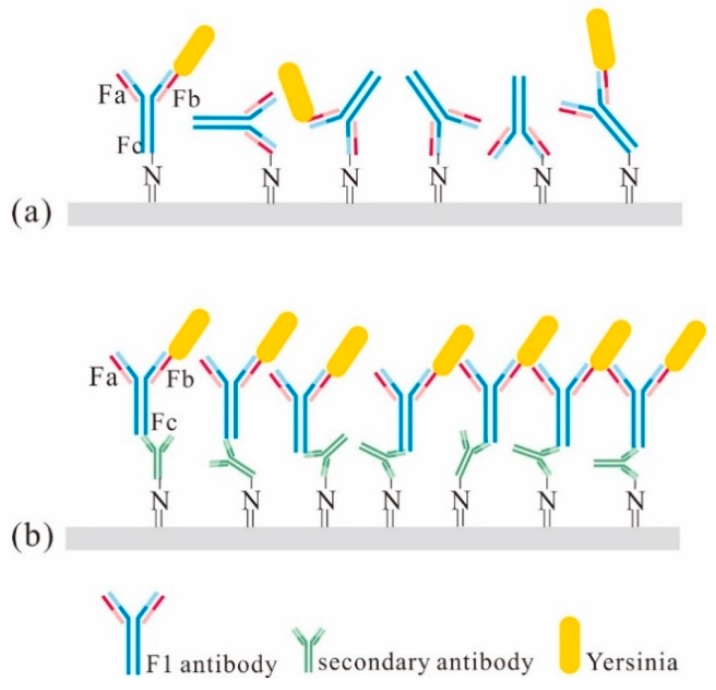
Microcantilever surface modification (**a**) without and (**b**) with a secondary antibody.

The reaction rate depends not only on the volume concentration at the wall but also on the available sites for adsorption. The net rate of adsorption is:

(3) $$\frac{d\Gamma }{\mathrm{dt}}=k_{on}\left(\Gamma_{0}-\Gamma \right)c_{0}-k_{\mathrm{off}}\Gamma$$

where *k_on_* and *k_off_* are the adsorption and dissociation rates and *c*_0_ is the concentration at the wall. Also, *k_on_* and *k_off_* are related to the nature of the target and the receptor and the reaction temperature. Therefore, increasing the effective binding sites on the adsorption target can enhance the binding efficiency. Figure 7b shows how the secondary antibody sequenced the F1 antibody and increased the effective binding sites. The principle is that the secondary antibody specifically reacts with the crystalline fragment (Fc) of F1, thereby exposing the effective binding sites or antigen-binding fragment (Fab) capture sites of the F1 antibody and increasing its binding efficiency. 

### 3.2. Yersinia Detection with Fully Immobilized Lever 

In order to verify the performance of the cantilever-based mass sensor, Yersinia solution at a concentration of 10^7^ cells/ml was prepared. To further ensure the accuracy of the test, there was a control test with PBS buffer solution. A 30 μL sample of PBS buffer solution or Yersinia analyte with the desired concentration was mixed on a vortex mixer for 1 min. The samples were injected into the microchannel and reacted with the cantilever immobilized with F1 antibody at 37 °C for 1 h. After each test, the biosensor was rinsed with PBS buffer solution and dried in the air. Images were taken with an optical microscope.

**Figure 8:**
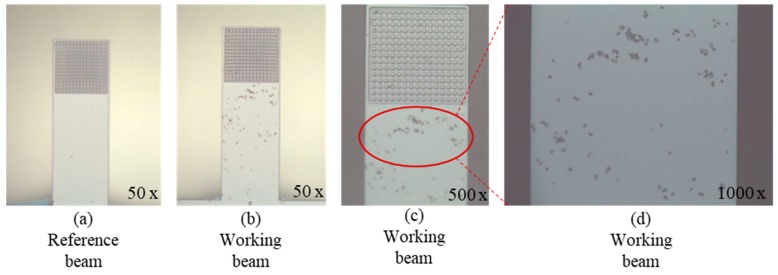
Microscope pictures of static reaction results: (**a**) reference beam; working beam at (**b**) 50×, (**c**) 500×, and (**d**) 1000× magnification.

Figure 8 shows the response of the biosensor, and the reaction was carried out under static conditions. The experiments were repeated three times, and the average value and standard deviation were used. The frequency shift was 37 Hz for the working beam and 7 Hz for the reference beam. The microscope pictures in Figure 8a,b clearly show that there is no adsorption of Yersinia on the reference beam, while for the working beam, special adsorption of Yersinia by F1 antibodies on the surface of cantilever is obvious. However, Figure 8c with the 500× magnification shows that in the microreaction cavity, comparatively few bacteria were observed, and most of the adsorption occurred in the surface area without the micropillars, as shown in Figure 8d, which was not expected. This may be related to the small space between the microcolumns. In static state, the analyte could not fully contact with the functioned microcolumns, so F1 antibodies could not effectively capture the Yersinia.

For the micron-sized particles in liquid solution, in the static case, diffusion caused by Brownian motion was mainly considered [21]. For a particle diffusing a distance *d*, the needed time can be expressed as:

(4) $$d∼\sqrt{Dt}$$

where *D* is the diffusion coefficient, 10^−10^ cm^2^/s magnitude for Yersinia [22], which suggests that Yersinia diffuse at a speed of around 1.2 μm/s. When the analyte concentration is small, this distance is not long enough compared to the dimensions of the entire reaction chamber, especially when the reaction time is short.

Therefore, the microfluidic cantilever system was applied to improve the capture efficiency of Yersinia with microfluidic flow control devices such as syringe pumps or peristaltic pumps. The syringe was installed in the peristaltic pump and reaction liquid flowed into the reaction chamber at a rate of 1 mL/min. Both the Yersinia test and control test were performed in the microchannel platform.

**Figure 9:**
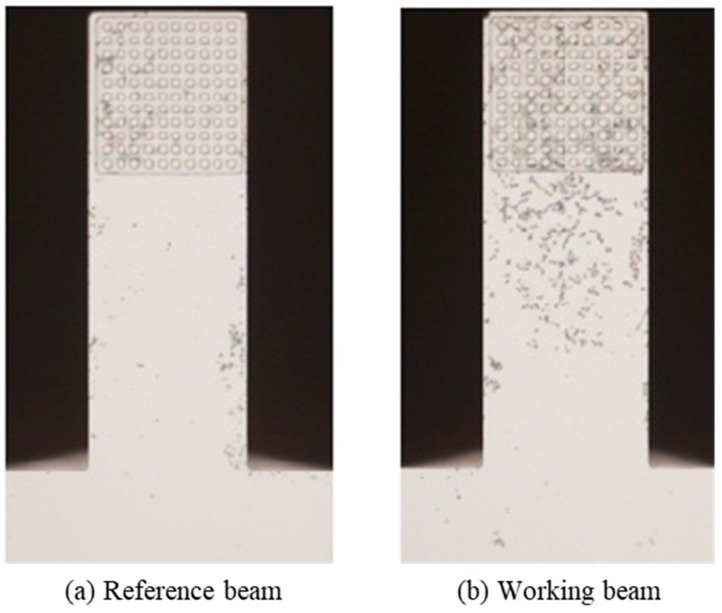
Detection results of Yesinia with microfluidic platform at a flow rate of 1 mL/min: (**a**) reference beam and (**b**) working beam.

Figure 9 shows that for the reaction solution with 10^7^ concentration bacteria, the whole beam adsorbed a large number of Yersinia. Moreover, the amount of adsorption significantly increased in comparison with the static experiments (Figure 8). The frequency shift for the working beam was 364.9 Hz. The frequency variation of the working beam was 10 times that of the previous one (37 Hz), where the reaction efficiency was greatly improved. However, in the corresponding control experiment, there were residual Yersinia bacteria on the microcolumn region of the reference beam, and the frequency shift was 148.6 Hz. This was nonspecific adsorption and was most likely due to the hydrophobicity of the bacteria, which causes it to agglomerate at the edge of the microcolumns under capillary force. For this reason, there was still residue after the cleaning process. By subtracting the frequency change of the reference beam from the working beam, there is still a frequency change of 216.3 Hz, where the reaction efficiency is greatly improved. A systematic study on the detection of Yersinia pestis is still in process.

## 4. Conclusions

This paper presents a method of Yersinia detection with a novel highly sensitive microcantilever array sensor. The importance of enhancing binding sites was theoretically analyzed, and the second antibody was introduced to increase capture efficiency. By comparing the adsorption of the same concentration of Yersinia on the surface of the microcantilever in both the static state and the flowing state, the importance of controlling the reaction velocity through the microfluidic platform was illustrated.
